# Illusory drifting within a window that moves across a flickering background

**DOI:** 10.1068/i0695sas

**Published:** 2014-11-20

**Authors:** Stuart Anstis, Sae Kaneko

**Affiliations:** Department of Psychology, University of California—San Diego, La Jolla, California; e-mail: sanstis@ucsd.edu; Japan Society for the Promotion of Science; Department of Psychology, University of California—San Diego, La Jolla, California; e-mail: sakaneko@ucsd.edu

**Keywords:** illusion, motion, position-shift, reverse-phi

## Abstract

When a striped disk moves across a flickering background, the stripes paradoxically seem to move faster than the disk itself. We attribute this new illusion to reverse-phi motion, which slows down the disk rim but does not affect the stripes.

Experiments on human motion perception have often used moving patterns that are viewed through a window. The window itself can be stationary or moving. Such motion within a window can affect the window's perceived position or motion. Anstis (1989) and De Valois and De Valois (1991) demonstrated a so-called “motion-induced position shift,” where perceived window position was biased toward the direction of motion within. The infinite regress illusion (Tse & Hsieh, 2006) and the Curveball illusion (Shapiro, Lu, Huang, Knight, & Ennis, 2010) showed that motion within a window also affects the window motion, especially in peripheral vision.

Here, we introduce a converse illusion, in which the window motion clearly affects the pattern within (Figure 1). In Figure 1a, a striped disk moves bodily to the right. This is like a moving grating seen through a moving window, both moving at the same speed. On a static background, this stimulus is seen correctly. But on a flickering background, the grating appears to move noticeably *faster* than the window, apparently drifting to the right within a slower moving window (Figure 1b).

**Figure 1.**
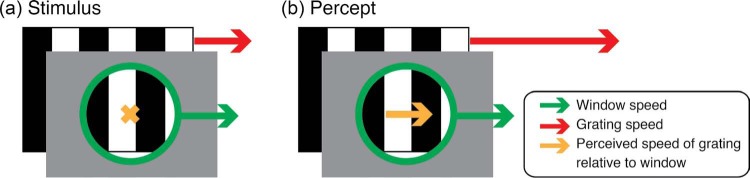
(a) A striped disk moves bodily to the right across a flickering background. The window and the grating behind it move at the same speed, but the grating appears to move faster than the window (b, long red arrow), apparently drifting to the right within a slower moving window (b, short green arrow).

Movie #1 shows this illusion. The upper, control disk is seen veridically, while the lower disk seems to contain a speeded-up grating, owing to the flickering background. A thin outline grey circle enhances the illusion. Random dots (not shown) inside the window give the same apparent speedup as gratings.

Movie #2 indicates the strength of this illusion. You can either track each moving window, or else fixate one of the four central labels to view the illusion peripherally. In the four control windows on the left, it is easy to see that the grating moves at 100% of the speed of the window where labelled 100%, and at 25%, 50% and 75% of the window speed where so labelled. But in the corresponding windows on the right, the flickering background makes the gratings appear to move much *faster* than the windows. For most observers, the gratings appear locked to the windows labelled 25% or 50%.

We measured this apparent increase in grating speed by a nulling method. The target stimulus was a 4°-diameter circular window, rimmed with grey and containing a 1.2 cpd grating. The window moved repetitively to the right through a distance of 12° at a speed of 6°/s. The background was either static uniform black, or flickering between black and white at 7.5 Hz. Observers either tracked the moving patch (foveal condition) or fixated a stationary point at ∼6° eccentricity (peripheral condition). They adjusted the speed of the grating using designated keys until it appeared to move at the same speed as the window (so that it looked like a moving striped disk). Observers made at least eight consecutive estimates.

Grating speed settings are shown in Figure 2. All setting speeds were below 100% (= physical window speed), which means that gratings appeared locked to their windows when they were really moving slower than the window. For instance, a grating viewed peripherally on a flickering background appeared to move in sync with the window when it was moving at 42% of the window speed (×2.4 relative speedup). Flickering backgrounds made the gratings look faster than the windows, as confirmed by a two-way ANOVA (*F*(1,4) = 27.40, *p* < .05). Peripheral viewing gave stronger effects than foveal viewing (*F*(1,4) = 16.35, *p* < .05).

**Figure 2.**
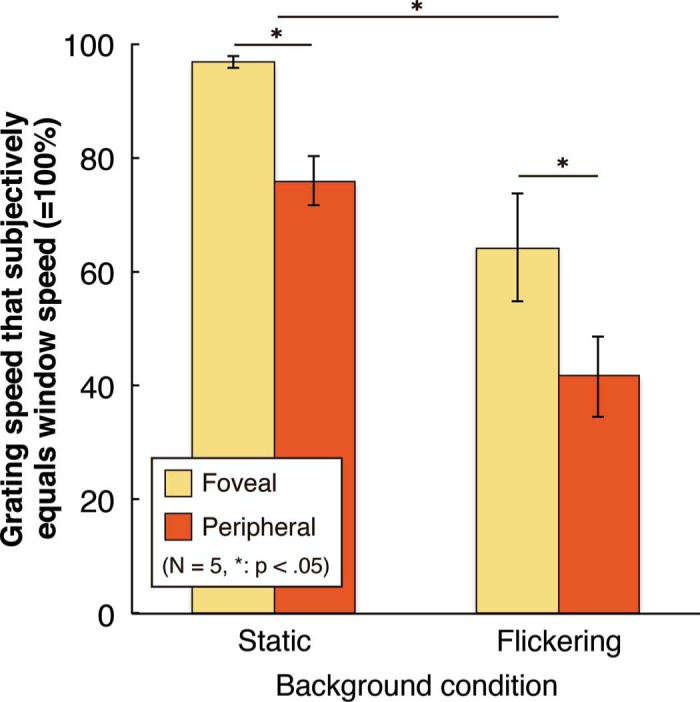
Average grating speeds (±1SE) that appeared to move at the same speed as the window; 100% equals physical window speed. Shorter bars indicate more slowing applied, hence bigger illusions.

Although the grating subjectively appears to speed up relative to the window, we attribute this illusion to the windows being slowed down by reverse-phi (Anstis, 1970; Anstis & Rogers, 1975). In reverse-phi, an object that continually reverses its contrast as it moves to the right appears to move to the left. Reverse-phi is thought to occur in simple cells in cortical area V1 (Livingstone & Conway, 2003). Reverse-phi is stronger in peripheral vision (Edwards & Nishida, 2004), which is consistent with our data.

The grey rim of every moving window in Movies #1 and #2 was alternately lighter and darker than its flickering surround and therefore reversed its polarity as it moved. But the gratings within, being spatially remote from the flicker, did not undergo reverse-phi, so they were not slowed down but appeared to move faster than the window. In further support of this reverse-phi hypothesis, a black rim instead of a grey rim abolishes the illusion (Movie #3), and a flickering surround slows down even an empty uniform grey disk (Movie #4). Also, the illusion disappeared when the background flicker was colour-defined. More demonstrations of this illusion can be found at our website (http://quote.ucsd.edu/anstislab/?p=1315).
